# Chemical Synthesis of Silk-Mimetic Polymers

**DOI:** 10.3390/ma12244086

**Published:** 2019-12-06

**Authors:** Amrita Sarkar, Alexander J. Connor, Mattheos Koffas, R. Helen Zha

**Affiliations:** Department of Chemical and Biological Engineering, Rensselaer Polytechnic Institute, Troy, NY 12180, USA; amritasrkr@gmail.com (A.S.); connoa5@rpi.edu (A.J.C.); koffam@rpi.edu (M.K.)

**Keywords:** silk-mimetic polymers, bioinspired materials, macromolecular self-assembly

## Abstract

Silk is a naturally occurring high-performance material that can surpass man-made polymers in toughness and strength. The remarkable mechanical properties of silk result from the primary sequence of silk fibroin, which bears semblance to a linear segmented copolymer with alternating rigid (“crystalline”) and flexible (“amorphous”) blocks. Silk-mimetic polymers are therefore of great emerging interest, as they can potentially exhibit the advantageous features of natural silk while possessing synthetic flexibility as well as non-natural compositions. This review describes the relationships between primary sequence and material properties in natural silk fibroin and furthermore discusses chemical approaches towards the synthesis of silk-mimetic polymers. In particular, step-growth polymerization, controlled radical polymerization, and copolymerization with naturally derived silk fibroin are presented as strategies for synthesizing silk-mimetic polymers with varying molecular weights and degrees of sequence control. Strategies for improving macromolecular solubility during polymerization are also highlighted. Lastly, the relationships between synthetic approach, supramolecular structure, and bulk material properties are explored in this review, with the aim of providing an informative perspective on the challenges facing chemical synthesis of silk-mimetic polymers with desirable properties.

## 1. Introduction

**Figure 1:**
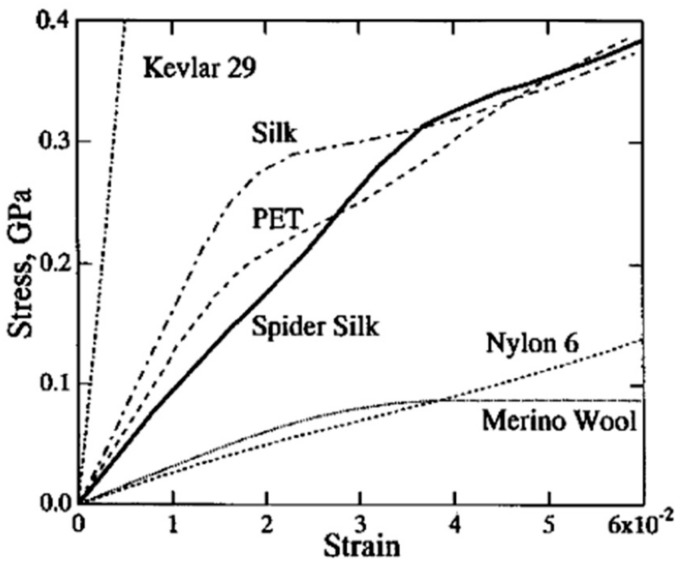
Tensile behavior of silk compared to various natural materials, including merino wool, silkworm silk (shown as “silk”), spider silk, and synthetic materials, including Kevlar 29, polyethylene terephthalate (PET), and nylon 6. The y-axis shows tensile stress (force per unit area) in GPa, and the x-axis shows tensile strain (extension normalized by initial length) as a dimensionless number. Copyright 2004 American Chemical Society, reproduced with permission from [44].

Silk is a protein-based material with extraordinary properties. Produced by many orders of arachnids and insects, such as Lepidoptera, Hymenoptera, Diptera, Neuroptera, and Araneae, silk functions as a structural fiber spun on demand for applications ranging from prey capture to egg encasement. For millennia, mankind has recognized silk as a highly robust natural material desirable for the fabrication of bowstrings, fishing nets, and clothing. To date, some of the most commonly studied silks have been derived from the cocoons of *Bombyx mori* silkworms, which have been domestically cultured for thousands of years for the production of silk textiles. More recently, silk threads from several orb-weaver spider species, including *Araneus diadematus* and *Nephila clavipes,* have been studied extensively. Specifically, dragline silk produced by the major ampullate gland of these spiders is responsible for the robust framework of spider webs and has been shown to exhibit mechanical properties that are unmatched by almost any other man-made or natural material [1,2,3]. With only a fraction of the density of steel, dragline silk can surpass even high-performance materials such as carbon fiber and Kevlar in toughness. This toughness results from a relatively high ultimate tensile strength combined with excellent extensibility, allowing dragline silk fibers to absorb the energy of high impact collisions from large prey. In comparison to man-made polymers, the combination of strength, toughness, and stiffness exhibited by silk is unmatched (Table 1 and Figure 1) [2].

Aside from outstanding mechanical properties, silk is also biodegradable, thermally stable until 285 °C [4], and resistant to dissolution most organic solvents [1,5]. The properties of silk, such as strength and modulus [6], glass transition temperature [7], degradation rate [8], and macromolecular conformation [9], can be tuned by controlling environmental and processing conditions. Furthermore, silk can exhibit good biocompatibility and illicit minimal inflammatory response in vivo [10,11,12,13,14,15]. Thus, naturally derived silk has been utilized extensively as a primary component in commercially available surgical sutures. Studies have also demonstrated the ability to process silk into non-woven mats, films, coatings, hydrogels, microcapsules, and sponges for various biomedical applications ranging from tissue regeneration to drug release [16,17]. Moreover, recent studies have revealed that silk fibers can exhibit interesting optical properties, such as the potential for pristine *N. clavipes* dragline silk [18] and *B. mori* cocoon silk (with outer sericin protein layer removed) [19] to act as natural waveguides. Similarly, *B. mori* silk fibroin films can also act as planar waveguides for visible and near-infrared wavelengths [20]. Combined with responsiveness of silk fibroin materials to stimuli, such as humidity, these photonic properties enable the development of silk-based optical sensors [21]. Researchers have also shown that silk can be processed by inkjet printing [22], soft lithography [23], and nanoimprinting with and without readily incorporated fluorophores [24,25] to form novel nano/micro-patterned protein-based photonic materials, such as active optofluidic devices [26]. Extensive discussion of these recent research efforts has been presented in several seminal review articles [27,28] but is beyond the scope of our current review, which will focus on discussing methods and perspectives relevant to the chemical synthesis of silk-mimetic polymers. 

The rise of modern techniques in genomics, biochemistry, and soft matter materials science in recent decades have enabled a detailed study of silk from molecular to bulk length scales and from protein synthesis to extrusion. Many excellent reviews have been published on structure–property relationships in silk as well as the application and synthesis of silk-based materials [1,16,17,29,30,31,32,33,34,35,36]. Increasingly, attention has turned towards the rational synthesis of silk-mimetic materials with tunable properties to meet the continually expanding set of materials challenges for advanced material manufacturing, healthcare, and sustainability. To date, the synthesis of silk-mimetic macromolecules has primarily relied on recombinant production of proteins that mimic silk fibroin, the primary component of silk threads. For example, researchers recently demonstrated the production of a native-sized recombinant spider silk protein with mechanical properties comparable to *N. clavipes* dragline silk [37,38]. However, recombinant synthesis of silk-like proteins still faces challenges, including difficulties in creating repetitive gene constructs and expressing proteins with high glycine content. As a result, the size, sequence diversity, and production scale of recombinant silk-mimetic proteins remain somewhat limited. For further discussion on the recombinant production of silk-mimetic materials, we direct the reader to several hallmark reviews in the field [39,40,41,42,43].

In contrast to recombinant synthesis, chemical synthesis of silk-mimetic polymers is a relatively less explored strategy that may readily offer scalability and macromolecular tunability, including the introduction of non-natural polymeric backbones, which expands the functional space of silk-mimetic materials. This review will focus on the past, current, and future of chemical synthesis as a method for generating silk-mimetic materials. To provide context and aid readers who are unfamiliar with silk as a polymeric material, we will first provide an overview of structure–property relationships in natural silk. We will then discuss the current state-of-art developments in chemical synthesis of silk-mimetic polymers and summarize the principal challenges facing the field, providing outlooks towards future developments.

## 2. Structure–Property Relationships in Silk

### 2.1. Primary Sequence of Silk Fibroin

**Figure 2:**
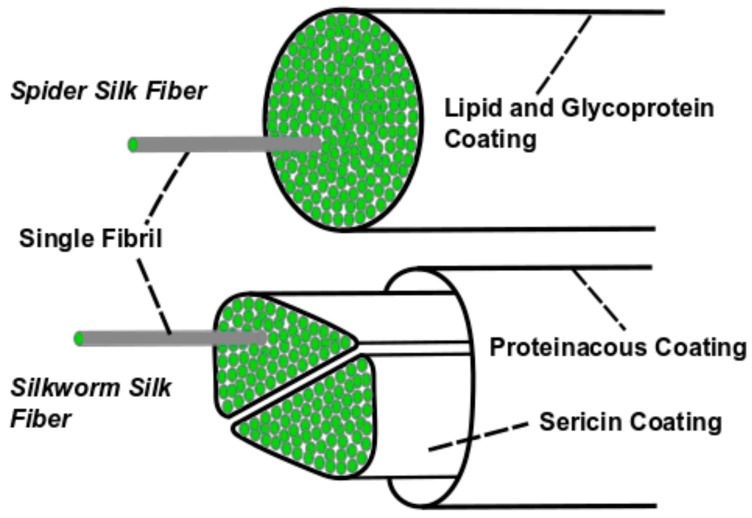
Mature silk threads consist of double silk fibroin cores coated by sericin (for silkworm silk) or a single spidroin core coated by lipids and glycoproteins (for spider silk). Importantly, silk fibroin and spidroin form nanofibrillar subunits within the cores.

The properties of silk fibers arise from chemical and structural interactions at hierarchical length scales. Silk threads are spun from the body of an organism as fibers that are several microns thick. The cross-sectional core of these fibers consists of silk fibroin, a class of structural proteins primarily responsible for the properties of silk materials. Spider dragline silk fibers contain one silk fibroin core coated by lipids and glycoproteins, while *B. mori* cocoon silk fibers contain double silk fibroin cores coated by glue-like sericin proteins (Figure 2). These cores are in turn comprised of many high aspect ratio silk fibroin nanostructures formed by supramolecular assembly, often referred to as silk fibrils.

**Figure 3:**
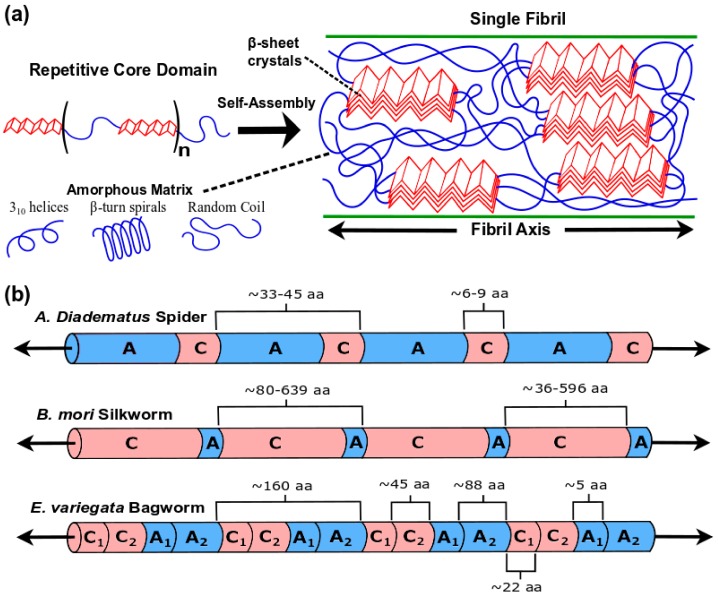
(**a**) Schematic of silk fibroin primary sequence, showing a repetitive core domain consisting of alternating rigid and flexible blocks. Self-assembly of silk fibroin results in the formation of nanocrystals, which consist of “stacked” β-sheets with hydrophobic interactions between amino acid side chains extending orthogonal from the sheet plane, embedded in a hydrophilic amorphous matrix. (**b**) Silk fibroin from different species exhibit varying primary sequences, with corresponding differences in mechanical properties. *A. diadematus* ADF-4 dragline spidroin consists of alternating flexible “amorphous” (A) and rigid “crystalline” (C) blocks, where C is 6–9 alanines and the A-C length is typically 33–45 amino acids (aa) long. *B. mori* silk fibroin consists of A-C repeats ranging from 80–639 amino acids where C is rich in glycine-X motifs. *E. variegata* silk fibroin consists of A-C repeats approximately 160 amino acids long, where C and A blocks are divided into 2 distinct sections. C_1_ specifically consists of a (A)_9_E(A)_12_ sequence, while C_2_ and A_2_ are glycine-rich. A_1_ is a short linker rich in valine.

The primary sequence of silk fibroin drives the structure, properties, and functionality of silk. In recent decades, genomic sequencing has elucidated the whole or partial primary sequence of various silk fibroin, most notably from *B. mori* cocoon silk [45] as well as the dragline silk of several spider species [46,47]. Although silk fibroin primary sequence varies depending on the properties and natural purposes of the silk material, a linear architecture that consists of non-repetitive N and C-terminus domains and a highly repetitive core domain is generally observed (Figure 3a). While the non-repetitive termini may play a role in initiating protein assembly during spinning [48,49,50], the core domain accounts for the majority of the protein sequence and is crucial to the mechanical properties of silk [3]. Generally speaking, the repetitive core domain is a polypeptide chain consisting of rigid hydrophobic segments, which form inter- and intra-chain nanocrystals, alternating with flexible hydrophilic segments, which form an amorphous matrix (Table 2 and Figure 3). For example, *B. mori* silk fibroin is comprised of a 391 kDa heavy chain linked to a 26 kDa light chain by a disulfide bond [40]. The heavy chain, which dominates the mechanical properties of *B. mori* silk, has a repetitive core domain with 12 rigid segments bearing long stretches of glycine-X (GX) repeats, where X is most commonly alanine (A), serine (S), or tyrosine (Y) (Figure 3b). These GX repeats favor antiparallel β-sheet secondary structure, which have a further tendency for assembly into crystalline domains detectable by characteristic Bragg peaks in X-ray diffraction [51,52]. In particular, GAAS, GAGAGS, and GAGAGY are commonly encountered motifs in these rigid segments. The 11 flexible linker segments between the rigid segments are nearly identical in sequence and include a non-repetitive 25 amino acid sequence that, notably, contains the proline (P), which generally disrupts secondary structure [53], and glutamic acid (E), which is negatively charged at physiological pH. 

The primary sequence of spider silk fibroin (spidroin) is qualitatively similar to that of *B. mori* silk fibroin as described above. Dragline silks are composed of two different spidroins, such as major ampullate 1 (MaSp1) and major ampullate 2 (MaSp2) spidroins produced by *N. clavipes* [54]. Similarly to the silk fibroin heavy chain, the repetitive core domain of many spidroins comprises over 90% of the protein and consists of alternating rigid and flexible segments. One rigid-flexible “diblock” unit is typically 10–50 residues long and may repeat several hundred times in the core domain. Unlike *B. mori* silk fibroin, the rigid segments of dragline spidroins often contain 6–9 tandem alanine residues instead of GX motifs. These oligoalanine stretches form β-sheets with methyl side chains extending above and below the sheet plane. Hydrophobic interaction of the methyl side chains then drives the “stacking” of β-sheets into strong and rigid supramolecular nanocrystals approximately 2–20 nm in size, though it should be emphasized that this differs from aromatic π–π stacking commonly discussed in chemistry and structural biology texts. In contrast, the GX motifs of *B. mori* silk fibroin have exposed methyl groups on only one face and may thusly exhibit relatively weaker “stacking”, leading to lower tensile strength, stiffness, and toughness in comparison to dragline silk (Table 1). Furthermore, the flexible segments of dragline spidroins often contain multiple repeats of GPGXX and GGX motifs, where X is commonly tyrosine (Y), glutamine (Q), or leucine (L) [5,55,56]. These motifs are prone to forming 3_10_ helices and β-turn spirals, which contribute elasticity that works in conjunction with the stiffness and strength provided by the nanocrystals. Interestingly, flagelliform spidroin produced by spiders for the capture spiral of their webs consists almost entirely of GPGXX (87%) and GGX (11%) motifs [55,56], resulting in materials with high toughness and failure strain.

The commonality of certain motifs in various silk fibroins point towards the “modular” nature of silk fibroin primary sequence. Proper combination of modules can have dramatic effects on material properties. Studies recently found that fibroin contained in silk threads produced by *Eumeta variegata* (Japanese bagworm) exhibit GX, GGX, and oligoalanine motifs, thus combining elements observed in spider and silkworm fibroins. In particular, long segments (~160 residues) consisting of the oligoalanine sequence (A)_9_E(A)_12_ in tandem with a sequence rich in GX and GGX was found to repeat five times in this fibroin. Notably, the oligoalanine sequence is more than double the length of those found in dragline spidroin, with the glutamic acid residue most likely acting to aid solubility. Consequently, *E. variegata* silk fibroin exhibits highly crystalline nanodomains and far surpasses dragline silk in stiffness, strength, and toughness.

### 2.2. The Supramolecular Nature of Silk

Silk is a supramolecular polymer [2], which is defined here as a macromolecular material with properties that are significantly influenced by non-covalent intermolecular interactions. While di-tyrosine crosslinking via oxidation and lysine-mediated crosslinking by hydroxylation may occur [60], non-covalent interactions predominantly account for the cohesive forces within silk materials. Aside from physical entanglements of silk fibroin polypeptide chains, hydrogen bonding and hydrophobic interactions play significant roles in determining the mechanical properties of silk. As described in the previous section, nanocrystals formed by β-sheet “stacking” act as supramolecular crosslinks as well as rigid domains that reinforce an elastomeric network. This supramolecular structure underlies the strength and robustness of silk, and several models have been developed to describe the response of silk to mechanical stress [61,62,63,64,65]. Furthermore, the non-covalent interactions of silk fibroin enables self-healing, most notably demonstrated in nature by the phenomenon of supercontraction, wherein spider silk threads swell in high humidity to “plasticize” and repair the supramolecular network [6]. 

The transition of silk fibroin from an unstructured, soluble protein to a robust β-sheet-rich material is of significant curiosity to supramolecular and biomaterials researchers. Changes in pH, ion composition, and water content are known to occur as the spinning dope passes through the spinning duct [66]. For example, a pH gradient of >7 in the posterior silk gland to <6.5 in the anterior silk gland has been found in *B. mori* silkworm larvae and in several spider species [67,68,69]. Furthermore, increasing phosphate ion and decreasing sodium chloride levels have been observed along the major ampullate spinning ducts of spiders [68], while microvilli lining the lumen of the anterior silk gland are thought to absorb water molecules from the dope [70]. The overall effect of these factors is to promote rapid β-sheet assembly and supramolecular aggregation. Several competing hypotheses exist for the specific mechanisms of silk fibroin structural transition in vivo, on which the reader is referred to relevant literature for further discussion [2,71,72,73,74,75]. Importantly, the non-repetitive N and C-terminus domains have been implicated in controlling silk fibroin self-assembly [48,49,50]. However, studies of recombinant proteins that mimic only the repetitive core domain of dragline spidroins have shown that β-sheet-rich materials can form without these non-repetitive domains in response to decreasing pH, increasing the content of structure-promoting kosmotropes such as phosphate ions, or elevated temperature [76,77,78]. Under certain solution conditions, the formation of nanofibers or globular nanostructures by recombinant as well as native silk fibroin can be observed [79,80,81].

### 2.3. Silk Fibroin as a Polymer

From the perspective of a polymer chemist, silk fibroins most closely resemble linear segmented copolymers, such as poly(ether-*block*-amide), which are thermoplastic elastomers that exhibit crystalline polyamide domains dispersed in an amorphous polyether matrix. However, silk can far surpass such commercially available thermoplastic elastomers in strength and stiffness [2]. This discrepancy in mechanical properties has not yet been explored. The volume fraction, length, and uniformity of β-sheet forming segments are expected to effect material properties, and these parameters in silk fibroin are known to vary amongst species. For example, the repetitive core domains of *N. clavipes* and *A. diadematus* dragline spidroin consist of rigid-flexible diblock repeats each approximately 40 residues long (~3.2 kDa), where the rigid oligoalanine stretch constitutes approximately 10% by mass [46,47]. In comparison, the rigid GX segment in *B. mori* silk fibroin heavy chain comprises over 50% of the protein by mass [45]. As discussed in Section 2.1, it is known that varying silk fibroin sequences give different mechanical properties (Table 1). However, the quantitative impact of macromolecular parameters in silk-like materials has not yet been examined in a systematic manner. Nevertheless, it is generally understood in man-made segmented copolymers that such parameters should play a significant role in determining mechanical properties. For example, Young’s modulus and ultimate tensile strength increases with the volume fraction of the rigid, nanocrystal-forming segment while maximum strain decreases. However, even in man-made amide-containing copolymers, structure–property relationships are not well understood, as polymerization strategies to date have produced ill-defined macromolecules [82]. Thus, with regards to the chemical synthesis of silk-mimetic polymers, strategies that can provide cost-effective sequence control and macromolecular tunability will likely be required.

## 3. Synthetic Approaches for Silk-Mimetic Segmented Copolymers

The synthesis of silk-mimetic materials has primarily utilized recombinant strategies to produce protein constructs that resemble the repetitive core domain of silk fibroin. For example, Scheibel and coworkers have published extensively on the recombinant synthesis and supramolecular characterization of eADF4(C16), a 47 kDa protein consisting of 16 repeats of a 35-residue consensus sequence mimicking the repetitive segment of *A. diadematus* dragline spidroin [81,83]. Zhang and coworkers have also worked towards the recombinant synthesis of silk-mimetic proteins, recently demonstrating the synthesis of a protein containing 192 repeats of a 35-residue *N. clavipes* dragline spidroin sequence [38]. Despite significant progress in the past decade, recombinant synthesis of silk-like proteins still faces technical challenges, including difficulties in creating and sequencing repetitive gene constructs with high guanine/cytosine (GC) content, expressing glycine-rich proteins in common *E. coli* strains, and preventing truncations or segment deletions during expression of high molecular weight proteins [84]. Extended discussion of recombinant synthesis of silk-mimetic proteins is out of the scope of the present review, but have been presented elsewhere [39,42,85,86]. Compared to chemical synthesis approaches, the sequence diversity of recombinant silk-mimetic proteins is somewhat limited, as recombinant synthesis cannot readily produce polymers with non-natural polymeric backbones which may be desirable for specific materials needs. Work on the chemical synthesis of silk-mimetic polymers has been sparse to date. We exclude, in our discussion, work regarding diblock or triblock architectures, where some progress has been made in designing self-assembling molecules driven by β-sheet formation [87,88,89,90,91,92,93,94]. We will limit our scope to silk-mimetic linear segmented copolymers containing many repeats of a rigid (“crystalline”)-flexible (“amorphous”) diblock, as this architecture can form the supramolecular elastomer network necessary for high mechanical robustness. As discussed in Section 2, the properties of silk are significantly impacted by macromolecular parameters such as peptide sequence and molecular weight. Achieving sequence control and high molecular weight has been the primary challenge in the chemical synthesis of silk-mimetic polymers. In this section, we will discuss the work to date towards the chemical synthesis of silk-mimetic polymers, which is generally encompassed by three distinct synthetic strategies: I) chain extension and step-growth polymerization, II) controlled radical polymerization, and III) copolymerization with naturally derived silk fibroin. To emphasize the importance of structure–property relationships in silk-mimetic materials, we will also present relevant results on secondary structure formation and materials properties as part of our discussion.

### 3.1. Strategy I: Chain Extension and Step-Growth Polymerization

#### 3.1.1. PEG-Peptide Copolymers

**Figure 4:**
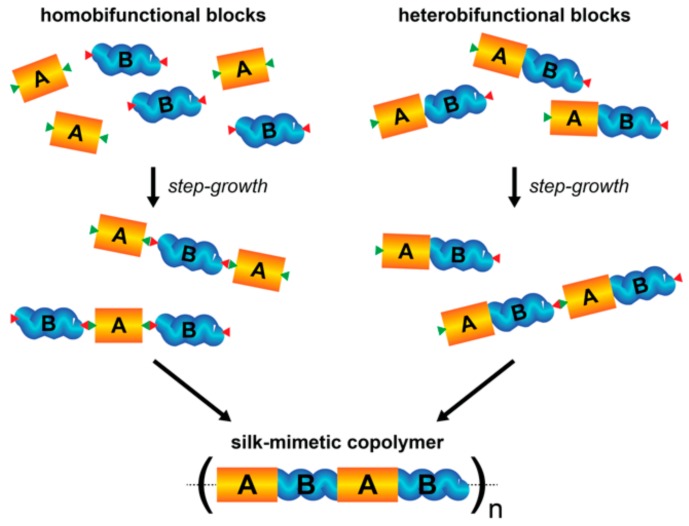
Schematic of step-growth polymerization using homobifunctional (left) or heterobifunctional (right) crystalline and amorphous prepolymers to synthesize silk-mimetic copolymers.

Step-growth polymerization is a common synthetic strategy where bifunctional or multifunctional monomers link to form progressively larger species (Figure 4). Segmented copolymers are often synthesized by connecting oligomers bearing reactive end groups, called prepolymers, with telechelic linkers, called chain extenders. This type of step-growth polymerization can accommodate a variety of coupling chemistries and does not require an initiator. However, a strict 1:1 stoichiometric balance of reactive end groups and an absence of side reactions that do not result in chain coupling are prerequisites for obtaining polymers with a high molecular weight. Moreover, step-growth polymerization inherently results in polymers with broad molecular weight distribution (Đ > 2 based on the Carothers equation). 

**Figure 5:**
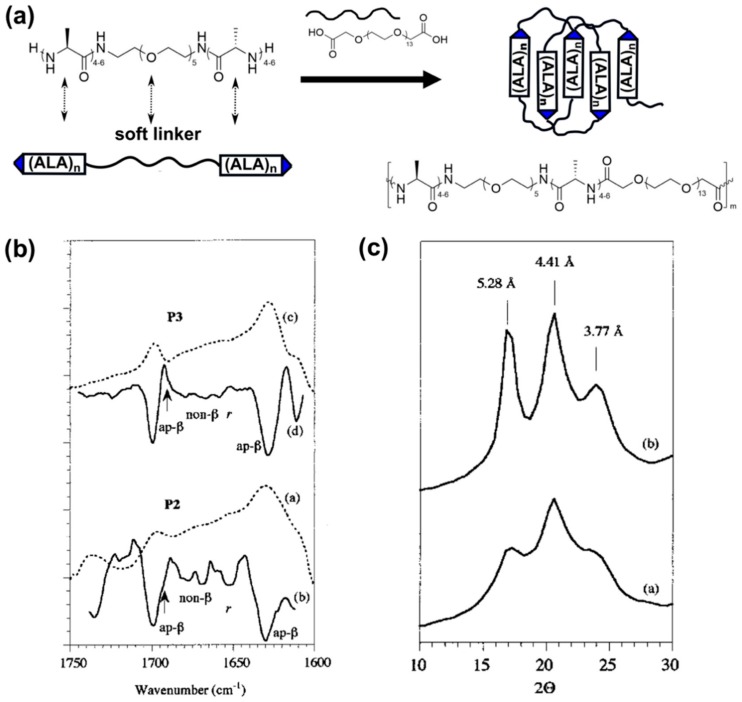
(**a**) Synthetic strategy used by Sogah and coworkers [96] to generate silk-mimetic polymers based on chain extension of an oligoalanine-PEG-oligoalanine triblock. Self-assembly of silk-mimetic polymers into β-sheet rich crystalline domains as shown by (**b**) solid-state FTIR and (**c**) powder XRD. FTIR spectra were obtained for P2 and P3, which are silk-mimetic ALA-PEG polymers were the ALA degree of polymerizations are approximately 4 and 6, respectively. P2 and P3 film samples were made by drop-casting from a 40% w/v solution dissolved in hexafluoroisopropanol. Resolution-enhanced spectra are shown (dotted lines a and c), as well as second derivative spectrums (solid lines b and d), showing the presence of anti-parallel β-sheets based on bands at 1630 and 1692 cm^−1^. Powder XRD of P2 (solid line a) and P3 (solid line b) shows *d* spacings of 5.28, 4.41, and 3.77 Å, which are consistent with anti-parallel β-sheet spacings reported for *N. clavipes* silk. (**b**) and (**c**) are Copyright 2001 American Chemical Society, reproduced with permission from [96].

Nevertheless, exploiting the simplicity of step-growth polymerization, Sogah and coworkers first demonstrated the synthesis of silk-mimetic copolymers based on oligoamide and poly(ethylene glycol) (PEG) building blocks [95,96,97]. Here, PEG was chosen as a substitute for the flexible peptide segment of silk fibroin based on its commercial availability and known biocompatibility [98]. Using α,ω-diamino-PEG as an initiator, oligoalanines (ALA) were synthesized using anionic ring opening polymerization (ROP) of N-carboxyamino acid anhydrides (NCA), resulting in disperse oligoalanine chains with average degrees of polymerization (DP) of 4 or 6 residues (Figure 5) [96]. This triblock prepolymer was then reacted with a bifunctional PEG-bis(carboxymethyl) ether (average M_n_ ~ 600 g/mol) chain extender to generate the ALA-PEG silk-mimetic copolymer. The step-growth polymerization was performed in dimethylsulfoxide (DMSO) with 2.5 wt% LiCl at room temperature for 4 days using diphenylphosphoryl azide (DPPA) and triethylamine (NEt_3_) as coupling reagents. In particular, DPPA was chosen as a coupling agent for amide bond formation based on its previously demonstrated utility in synthesizing peptide-polymer hybrids [99]. The addition of LiCl inhibits the aggregation of ALA segments during polymerization. Due to the high volume fraction of the ALA segments, these ALA-PEG copolymers exhibited extremely poor solubility in most organic solvents, including DMSO and dimethylformamide (DMF). Thus, in lieu of gel permeation chromatography, molar masses were studied by measuring inherent viscosity (η_inh_) in dichloroacetic acid (DCA). Although this synthetic strategy yielded moderately high molecular weights (M_w_ ~ 25 kg/mol obtained from η_inh_), the dispersity of the copolymers as well as the individual ALA segments were broad, potentially affecting secondary structure formation. However, the ALA-PEG copolymers did demonstrate silk-like microphase separated morphologies, with 100–200 nm domains rich in antiparallel β-sheets dispersed in a continuous PEG matrix [96,97]. Furthermore, longer ALA segments were observed to have a greater propensity for antiparallel β-sheet assembly, leading to increased material stiffness and tensile strength as well as decreased toughness. Importantly, it was found that films fabricated from these ALA-PEG copolymers had better stiffness and strength than films made from regenerated spidroin, and fibers fabricated from the ALA-PEG copolymers were comparable to natural silk threads in mechanical properties [96].

**Figure 6:**
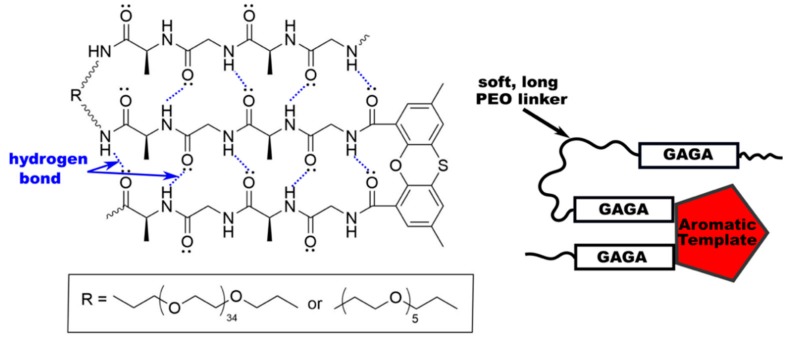
Synthesis of silk-mimetic polymers by Sogah and coworkers [97] with an aromatic hairpin to template parallel β-sheets.

In separate work, Sogah and coworkers explored two different synthetic approaches in order to study the effect of templated vs. conformationally flexible linkers on β-sheet formation in silk-mimetic copolymers [97]. Here, tert-butyloxycarbonyl (Boc)-protected GAGA tetrapeptides mimicking the rigid β-sheet forming segment of *B. mori* silk fibroin were conjugated to α,ω-diamino-PEG with short (M_n_ ~ 230 g/mol) or long (M_n_ ~ 1630 g/mol) chain lengths. The resulting triblocks were then conjugated to phenoxathiin hairpins such that the GAGA tetrapeptides were constrained in a parallel orientation (Figure 5) [95,97]. Alternatively, the triblocks were chain extended using PEG-bis(carboxymethyl) ether to generate a linear copolymer without the hairpin template (Figure 6). Coupling reagents such as DPPA and collidine with hydroxybenzotriazole and polar aprotic solvents such as DMSO, DMF, and N-Methyl-2-pyrrolidone (NMP) were used in these synthetic schemes. Compared to the ALA-PEG polymers previously discussed [96], these GAGA-PEG copolymers exhibited improved solubility in organic solvents due to the lower aggregation propensity of the tetrapeptide. Molecular weights of up to M_w_ = 25.7 kg/mol (found by GPC using PEO standards) were obtained after a 2-day step-growth polymerization with DPPA, and the resultant polymers furthermore demonstrated a high β-sheet content with and without the hairpin template. Moreover, the GAGA-PEG copolymers generally formed antiparallel β-sheets unless the parallel hairpin template was incorporated, which is consistent with the entropic inhibition of intramolecular hydrogen bonding in a parallel β-sheet arrangement for unconstrained chains [100]. Lower β-sheet content was found with the longer PEG chain [97], potentially due to a lower peptide segment volume fraction. The stiffness and strength of these GAGA-PEG copolymers were observed to be lower than that of nylon 6,6 and the ALA-PEG copolymer described previously. However, tensile testing revealed that GAGA-PEG copolymers with the flexible PEG linker instead of the hairpin template exhibited better strength and toughness, thus suggesting the specific importance of an anti-parallel β-sheet conformation. 

#### 3.1.2. Copolymerization using Isocyanate Groups

**Figure 7:**
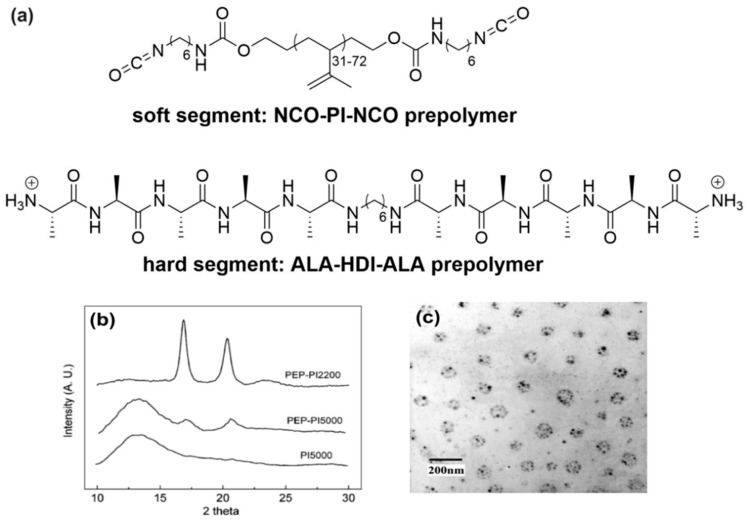
(**a**) Chemical structure of polyisoprene and oligoalanine prepolymers used by Shao and coworkers [101] to synthesize silk-mimetic polymers based on step-growth polymerization. (**b**) Self-assembly of these silk-mimetic polymers (PEP-PI2200) into β-sheet structures as demonstrated by characteristic sharp Bragg peaks in WAXD compared to silk-mimetic polymers with a longer polyisoprene chain (PEP-PI5000) or polyisoprene alone (PI5000). (**c**) Formation “plum blossom” micellar structures by self-assembly of these silk-mimetic polymers. (**b**) and (**c**) are Copyright 2006 American Chemical Society, reproduced with permission from [101].

The efficient formation of amide bonds by isocyanate and acid groups via an anhydride intermediate was explored by Shao and coworkers to synthesize silk-mimetic copolymers [101,102]. Here, Boc-protected glycine was conjugated to the ends of hexamethylene diisocyanate (HDI), and GAGA or GAAAA peptides were then grown from each end using stepwise solution-phase peptide synthesis, forming triblock prepolymers [102]. These prepolymers were then chain extended with HDI in DMSO, yielding segmented copolymers with repetitive peptide motifs mimicking the rigid β-sheet forming segment of *B. mori* silk fibroin (GAGA) or dragline spidroin (GAAAA). Molecular weights of 36.7–44.9 kg/mol (calculated from η_inh_ measurements) were achieved and both copolymers exhibited silk-like β-sheet secondary structures, as indicated by Fourier transform infrared spectroscopy (FTIR), wide-angle X-ray diffraction (WAXD), and ^13^C cross-polarization magic angle spinning NMR. Furthermore, Shao and coworkers pursued a similar synthetic strategy to synthesize silk-mimetic copolymers with AAAAA oligopeptides and polyisoprene (PI) amorphous segments of two different lengths (M_n_ = 2.2 kg/mmol and 5 kg/mol) (Figure 7) [101]. After polymerization for more than 2 days, a gel-like product was observed in the reaction vessel, which proved to be a β-sheet-rich segmented copolymer with poor solubility in most polar or nonpolar solvents, including hexafluoroisopropanol (HFIP). These synthesized polymers demonstrated the formation of antiparallel β-sheet structure. Furthermore, the copolymer with shorter polyisoprene blocks formed “plum blossom” micellar-like structures, whereas the same copolymer with longer polyisoprene blocks did not show nanostructure formation in transmission electron microscopy.

Step-growth polymerization using isocyanate groups has also been utilized to synthesize silk-mimetic thermoplastic polyurethanes (TPUs). Like silk fibroin, conventional TPUs consist of rigid (“hard”) and flexible (“soft”) segments that form a microphase segregated structure which enhances mechanical properties [103,104,105,106]. As TPUs contain urethane linkages rather than amide bonds, the incorporation of silk-like motifs introduces an interesting strategy for tuning materials properties [107,108,109]. In this work, GAGA tetrapeptides were synthesized stepwise in solution using Boc-protected amino acids from both ends of HDI [107]. The resulting triblock prepolymer was deprotected to reveal terminal primary amines and copolymerized with 4,40-methylene diphenyl diisocyanate-co-polytetrahydrofuran (pTHF, M_w_ ~ 2 kg/mol). The polymer product then was formed into granules by dropping the reaction solution into a 3:1 mixture of methanol and water, and further extracted in toluene [107]. Molecular weights of 13 kg/mol were achieved, which is slightly lower than the molecular weight of copolymers made in similar conditions with 1,4 butanediol instead of the GAGA-HDI-GAGA triblock [108]. Unlike silk fibroin and other silk-mimetic polymers previously described, these pTHF-containing silk-mimetic polymers demonstrated good solubility in a variety of organic solvents, which facilitated processing into fibers and films. Moreover, these silk-mimetic polymers showed evidence of antiparallel β-sheet conformation, despite containing urea and urethane linkages alongside amide bonds as part of the polymer backbone [107,108]. Similar results were recently obtained by Hu and coworkers, who demonstrated the synthesis of peptide-poly(tetramethylene ether glycol) (PTMEG) copolymers mimicking spider aciniform silk [110]. These copolymers were made by reaction of amine-terminated poly(γ-benzyl-*L*-glutamate) (PBLG) made by NCA ROP with isocyanate-terminated PTMEG, yielding β-sheet rich materials with high toughness.

#### 3.1.3. Copolymerization of Peptide with Semicrystalline Poly(ε-caprolactone)

**Figure 8:**
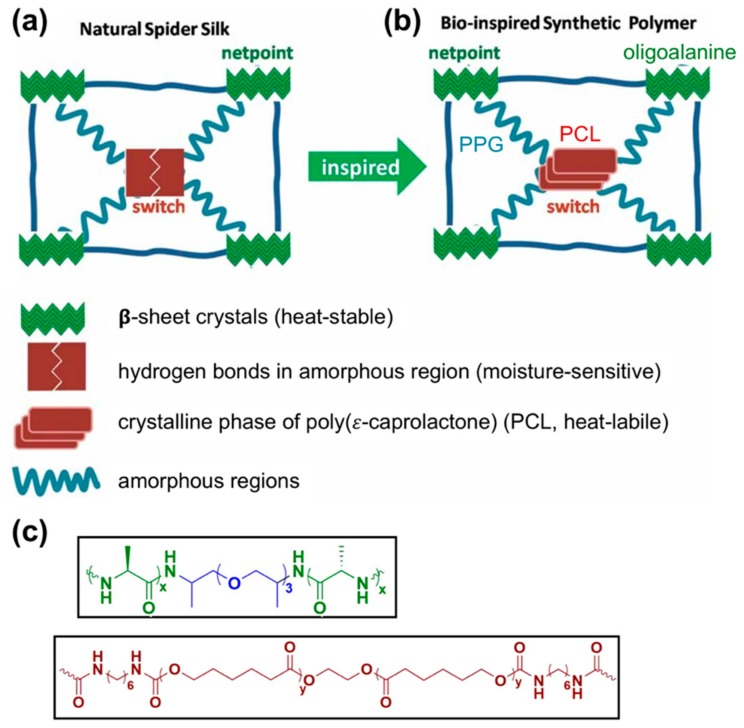
(**a**) Structural schematic of natural spider silk, which contains β-crystalline domains (green blocks) that act as heat-stable netpoints and hydrogen bonds (red dimer block) that act as moisture-sensitive netpoints, compared to (**b**) a synthetic bioinspired shape-memory polymer, which contains heat-stable silk-mimetic β-crystalline domains formed by oligoalanine and crystalline PCL domains (red rectangles) that act as heat-labile crosslinks. (**c**) The chemical structures of the PCL and oligoalanine-poly(propylene glycol) (PPG) segments of this bioinspired polymer are shown. While natural spider silk can undergo contraction and shape recovery when exposed to water or high humidity, these synthetic shape-memory polymers are temperature-sensitive, retaining a stretched state if cooled under tension and returning to their original shape upon re-heating. Copyright 2013 WILEY-VCH Verlag GmbH & Co, adapted with permission from [112].

While researchers synthesizing silk-mimetic polymers typically seek to capture the mechanical robustness of silk, the ability of spider dragline silk to undergo supercontraction (described in Section 2.2) in the presence of water or high humidity is also of interest to materials scientists. In particular, if silk is stretched while in a supercontracted state and subsequently dried, re-wetting the material can result in shrinkage to the original pre-stretched form [111]. Zhu and coworkers demonstrated that this shape-memory property can be achieved by synthesizing copolymers containing segments of oligoalanine and poly(ε-caprolactone) (PCL) (Figure 8) [112].

Here, the oligoalanine segments were synthesized by NCA ROP and had an average DP of eight residues (M_n_ ~ 1.3 kg/mol), while the PCL-diol was commercially purchased with M_n_ ~ 4 kg/mol. Using a two-step polymerization, the PCL-diol was first reacted with excess HDI in DMSO containing 2.5% LiCl at 85 °C for 1.5 h to prepare PCL-diisocyanate. Then, amine-terminated oligoalanine was added to the reaction for 6 h, yielding oligoalanine-PCL copolymers without sequence control. The synthesized polymers demonstrated the formation of crystalline oligoalanine domains rich in antiparallel β-sheets as well as crystalline PCL domains (Figure 8), as characterized by FTIR and WAXD techniques. Differential scanning calorimetry showed that the crystalline PCL domains had a melting temperature of 48–50 °C while the crystalline oligoalanine domains had no observable melting transitions below 280 °C, which is consistent with the high thermal stability of silk reported in literature [4]. Dynamic mechanical analysis of the polymers furthermore demonstrated that the polymers a rubbery plateau region above 50 °C, indicating that crystalline oligoalanine domains acted as network crosslinks independent of PCL domains. These separately addressable crosslinking domains endowed the polymer with shape-memory properties reminiscent to those of dragline silk, except that heat is used to “plasticize” the material instead of water. Materials that were stretched at 70 °C and cooled under tension were able to shrink to their original shape after re-heating.

#### 3.1.4. Chemoenzymatic Synthesis of Prepolymers

**Figure 9:**
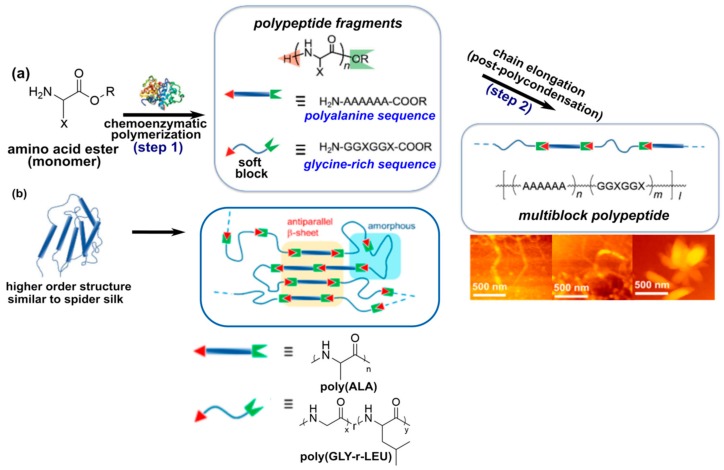
(**a**) Chemoenzymatic synthesis of polypeptide prepolymers for step-growth polymerization of silk-mimetic polymers. First, amino acid esters are used as monomers for papain-catalyzed polymerization of rigid polyalanine and soft glycine-rich polypeptides. Polycondensation is then used to synthesize silk-mimetic multiblock polypeptides. (**b**) Self-assembly of these polymers results in silk-like supramolecular structure, including antiparallel β-sheets and nanofiber morphologies visible by AFM. Copyright 2017 American Chemical Society, adapted with permission from [117]. Article and further information regarding permissions can be accessed at this direct link: https://doi.org/10.1021/acsmacrolett.7b00006.

Synthesis of peptide prepolymers for step-growth polymerization is typically achieved using stepwise amino acid coupling, which is relatively time-consuming and repetitive, or NCA ROP, which requires the use of toxic phosgene derivatives, organic solvents, and stringent reaction conditions to achieve peptides with low dispersity [113,114]. Chemoenzymatic polymerization is considered a “green synthetic method”, as reactions can be conducted in aqueous solvents with non-toxic bioderived reagents [115,116]. Moreover, chemoenzymatic polymerization overcomes the problem of epimerization by using enzyme catalysts with high reaction specificity. A comprehensive study on combining chemoenzymatic peptide synthesis with step-growth copolymerization to generate silk-mimetic polymers was reported by Numata and coworkers (Figure 9) [117]. Polyalanine and poly(glycine-*r*-leucine) peptides were synthesized by papain-catalyzed polymerization, followed by polycondensation of these peptides with polyphosphoric acid (PPA) as a coupling reagent. Here, the polyalanine and poly(glycine-*r*-leucine) segments were designed to mimic the rigid and flexible blocks of dragline spidroin, respectively. In particular, the polyalanine segment had a DP of 5–11, which is consistent with oligoalanine block lengths commonly found in native dragline spidroin. Unlike the step-growth polymerizations described previously, these peptide prepolymers were heterobifunctional, with an N-terminal amine and a C-terminal ethyl ester. Thus, in addition to having a disperse prepolymer segments, the rigid and flexible blocks in the resulting polymer were randomly sequenced. Furthermore, it was noted that the peptides exhibited poor solubility in common organic solvents; therefore, copolymerization at elevated temperatures and with NMP or dimethylacetamide (DMAc) as solvent was explored. Studies found that reaction in DMAc at 140 °C with 1 M LiBr for 2 days yielded the highest molecular weights. M_w_ of 17.2 kg/mol (measured by polystyrene-calibrated GPC) was achieved for a polyalanine-poly(glycine-*r*-leucine) feed ratio of 1:2, though the dispersity of this copolymer was unusually high (M_w_/M_n_ = 3.77, M_n_ = 4.6 kg/mol). Polymerizations in NMP did not achieve M_w_ higher than 5.2 kg/mol. Notably, all copolymers regardless of prepolymer feed ratio, solvent, or reaction conditions showed lower polyalanine content than expected, likely due to its lower solubility. WAXD analysis of the copolymers suggested that antiparallel β-sheet nanocrystals were present at 10%–20% crystallinity, which is comparable to the 15%–25% crystallinity found in native spidroin [3]. Furthermore, atomic force microscopy (AFM) showed that these polymers can form nanofiber structures similar to those observed in native and recombinant silk fibroin [77,80]. Thus, incorporation of peptides produced by chemoenzymatic synthesis is a promising approach towards the scalable synthesis of silk-mimetic polymers. However, further work is needed to overcome challenges, such as more precise sequence control as well as the inability to include proline, which is important to the elasticity of silk fibroin, due to its low affinity for papain [116].

### 3.2. Strategy II: Controlled Radical Polymerization

In the field of polymer chemistry, controlled or living radical polymerization (CRP) has proven to be a versatile technique for generating polymers with narrow dispersity from a variety of monomers [118]. Common CRP strategies include atom transfer radical polymerization (ATRP) [119], reversible addition-fragmentation (RAFT) [120], and nitroxide mediated polymerization [121]. The synthesis of several ABA or ABC-type triblock copolymers that mimic aspects of the silk fibroin primary sequence using ATRP have been reported [90,92,94,122,123], but examples demonstrating the synthesis of silk-mimetic segmented or multiblock copolymers using CRP techniques are rare. 

**Figure 10:**
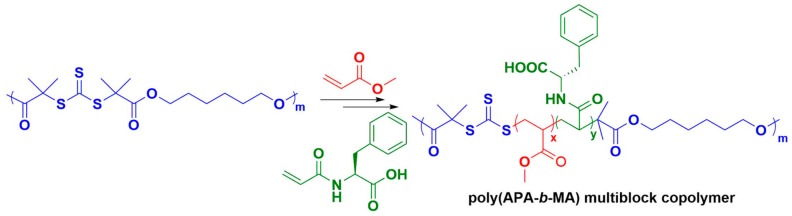
Synthetic strategy using polyfunctional trithiocarbonates as RAFT agents to synthesis silk-mimetic multiblock copolymers by sequential addition of methyl acrylate and *N*-acryloyl-*L*-phenylalanine monomers.

Indeed, the synthesis of (AB)_n_-type polymers by CRP is challenging but can be achieved using polyfunctional trithiocarbonates as RAFT agents (Figure 10) [124,125]. Vana and coworkers showed that polytrithiocarbonate (PTTC) can be used to synthesize poly(APA-*b*-MA), where APA is N-acryloyl-L-phenylalanine, which can form self-complementary hydrogen bonds, and MA is methyl acrylate, which forms a soft “amorphous” block [92]. In this strategy, PTTC is first synthesized from the bifunctional chain transfer agent *S,S*-bis(α,α′-dimethyl-α″-acetic acid)trithiocarbonate (DMATC). Then, RAFT polymerization of APA was performed using PTTC for 2 days at 60 °C in methanol. The resulting APA multiblock homopolymer was then purified by precipitation and used as a macromolecular RAFT agent for further polymerization of MA for 2 days at 60 °C. Based on initial studies of MA-APA-MA triblock copolymers, the ratio of APA to MA segment lengths in the silk-mimetic copolymer was adjusted to target maximum enhancement of mechanical properties. Poly(APA-*b*-MA) segmented copolymers were obtained with high molecular weight (up to M_n_ ~ 97 kg/mol, GPC with polystyrene standards). It was observed that these segmented copolymers had dispersity of up to Ð = 3.2, which is high compared to traditional RAFT polymerizations but is consistent with previous findings that polyfunctional RAFT agents yield much more disperse polymers than monofunctional RAFT agents [126]. It is also notable that in this synthetic strategy, the number of APA hard blocks and MA soft blocks is pre-determined by the DP of PTTC, which was calculated to be 9 on average [92]. Differential scanning calorimetry (DSC) of the poly(APA-*b*-MA) segmented copolymers showed possible evidence of microphase segregation, as two glass transitions corresponding to individual APA and MA blocks could be observed. However, no visual evidence of microphase segregation could be observed by AFM, possibly due to the small size of the APA segments compared to the MA segments. Uniaxial tensile tests showed that the poly(APA-*b*-MA) segmented copolymers had much higher ultimate tensile strength and toughness as well as more pronounced strain hardening compared to MA-*b*-APA-*b*-MA triblock copolymers, although these effects may result from higher molecular weight rather than from hydrogen bonding of the APA segments. IR spectroscopy was unable to discern hydrogen bonding in the segmented copolymers due to low signal intensity resulting from the low volume fraction of the APA segments compared to the MA segments. However, it was observed that samples drawn until failure could contract to their original dimensions after annealing at 100 °C (greater than the glass transition of the MA segments), thus suggesting the role of intermolecular hydrogen bonds in maintaining network cohesion.

### 3.3. Strategy III: Copolymerization with Naturally Derived Silk Fibroin

**Figure 11:**
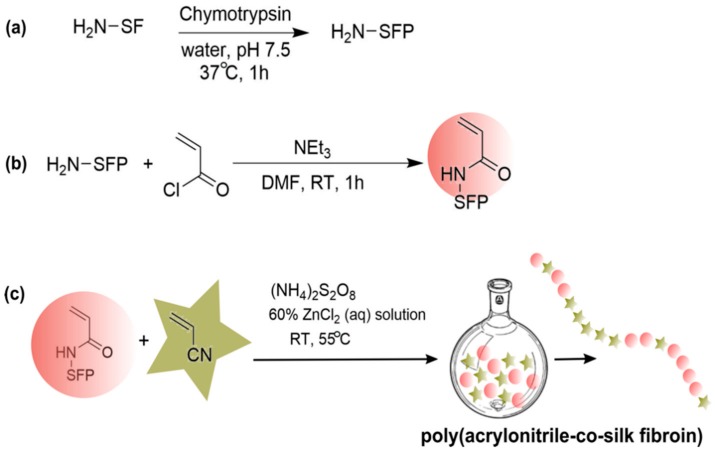
Multi-step strategy for the synthesis of silk-mimetic copolymers using naturally derived silk fibroin [128]. Here, (**a**) enzymatically fragmented silk fibroin (silk fibroin peptide, SFP) is (**b**) modified with vinyl acyl chloride and (**c**) then copolymerized with acrylonitrile.

As natural silk fibers show multiple material properties desirable in the textile industry, such as luster, mechanical robustness, and moisture absorption, silk fibroin extracted from *B. mori* cocoon silk is a promising starting material for further synthetic manipulation. Researchers have explored chemical modification of naturally derived silk fibroin, such as graft copolymerization of acrylonitrile (AN) onto the silk fibroin backbone in order to improve compatibility in acrylic polymer-silk blends [127]. However, in a unique approach towards generating silk-mimetic polymers, Shirai and coworkers utilized silk fibroin fragments as monomers for copolymerization with acrylonitrile (Figure 11) [128,129]. Here, silk fibroin was first extracted and purified from *B. mori* cocoons and digested with α-chymotrypsin, resulting in fragments ~25 kg/mol in size [128]. The N-terminus of silk fibroin fragments were then functionalized with vinyl acyl chloride, and copolymerization with AN was performed in aqueous conditions. This resulted in a copolymer that could leverage the beneficial properties of silk fibroin as well as the cost-effectiveness and photostability of polyacrylonitrile. Though no molecular weights were reported, successful polymerization was suggested by higher η_inh_ (4.9–9.5 dL/g). Interestingly, vinyl-functionalized silk fibroin monomers appeared to have higher reactivity during polymerization than acrylonitrile monomers. Copolymers with a silk fibroin content of 0.065, as measured by elementary analysis, were insoluble in DMF. Moreover, copolymers with the appropriate range of viscosities for fiber spinning were only achieved by adding Cu(II) and Fe(II) as chain transfer agents to hinder propagation of copolymer chains. These copolymers demonstrated higher thermal stability, with degradation occurring in the range of 285–290 °C compared to silk fibroin alone (degradation at 250 °C), although the copolymers exhibited lower thermal stability than polyacrylonitrile (degradation at 300 °C). Along with improved thermal stability, the copolymer demonstrated enhanced dyeability and moisture absorption compared to polyacrylonitrile and silk fibroin alone. FTIR confirmed the successful incorporation of silk fibroin fragments into the copolymer, although β-sheet formation was not discussed in these studies.

## 4. Challenges and Future Perspectives

Natural silk can exhibit multiple properties, such as thermal stability, mechanical robustness, and biocompatibility, which are highly desirable for advanced materials applications in human healthcare and sustainability. However, the pathway forward for leveraging the benefits of silk-based materials for real-world needs requires the rational synthesis of artificial silk-mimetic materials with tunable properties and cost-effective production methods. These synthetic efforts are aided by recent and ongoing research exploring the relationships between material properties and the molecular and supramolecular structure of silk. While recombinant synthesis of silk fibroin-like proteins has been the most common approach towards generating silk-mimetic materials, chemical synthesis can provide some advantages, such as the ability to incorporate non-natural backbones as well as potential scalability using established polymer synthesis infrastructure. We have thus far reviewed the progress to date in achieving silk-mimetic materials using a variety of chemical synthesis strategies. However, this field of work faces several significant challenges, including the poor solubility of silk-mimetic polymers, difficulties in obtaining polymers with high molecular weight, and unknown impact of sequence dispersity on materials properties alongside the practical complexities in achieving sequence control at scalable costs. 

Challenges with solubility are inherent to silk-based materials. Natural silk harvested after spinning is insoluble in water as well as most organic solvents due primarily to the robust intermolecular aggregation of segments into hydrophobic β-sheet nanocrystals. This insolubility complicates the processing of silk into useful forms and strongly chaotropic salts (e.g., 6 M guanidine thiocyanate) or acids that disrupt hydrogen bonding (e.g., hexafluoroisopropanol, trifluoracetic acid, formic acid, trifluorethanol) must be used to first disassemble the supramolecular structure of silk fibroin. Similarly, silk-mimetic polymers generally exhibit poor solubility in many organic solvents but can sometimes be dissolved in highly polar aprotic solvents such as DMSO, DMF, and DMAc (known solubilities for polymers are presented in Table 3). Insolubility increases with the volume fraction of the rigid “crystalline” peptide segment as well as with the inclusion of more hydrophobic amino acids. For example, the ALA-PEG copolymers reported by Sogah and coworkers were insoluble in most organic solvents, including DMSO and DMF, thereby preventing characterization by GPC [96]. However, when the tetrapeptide motif GAGA and a longer PEG chain was used, the resulting GAGA-PEG copolymer exhibited improved solubility in DMAc and DMSO [97]. Judicious choice of amorphous polymer segments, such as the use of pTHF instead of PEG [101,107], can also improve solubility in solvents such as THF and chloroform at the cost of forfeiting some silk-like material properties. Thus, creative solutions will be needed to synthesize polymers that fully mimic the properties of silk without suffering problems with solubility that hinder post-synthesis processing.

In recombinant silk fibroin materials, a high molecular weight has been demonstrated to be an important factor for strength, stiffness, and toughness [37]. This finding is consistent with the model of silk fibroin as an elastomeric network with physical entanglements and supramolecular crosslink “nodes” [61]. Longer molecules would result in more entanglements and chain connectivity between crosslinks, although it is not known whether improvements in mechanical properties become incremental above a certain chain length. However, the synthesis of silk-mimetic polymers with high molecular weight is challenging due to multiple factors. Firstly, while step-growth polymerization provides a facile and versatile route towards generating segmented copolymers, potentially with good sequence control, DP remains low unless high conversion can be achieved. In contrast, chain growth polymerizations can achieve high molecular weights at low conversions, although chain growth polymerization methods are not generally amenable for synthesizing copolymer architectures with many repeating segments. A second challenge with achieving high molecular weights lies in the aggregation of silk-mimetic copolymers as polymerization progresses. Many of the synthetic examples discussed thus far have utilized solvents such as DMSO, DMF, and DMAc with LiBr or LiCl added to reduce β-sheet aggregation during polymerization. Nevertheless, these solvents can limit the versatility of synthetic chemistries and are also difficult to fully remove from the polymer product, often requiring precipitation followed by lengthy dialysis. 

The role of dispersity and the importance of sequence control in silk-mimetic macromolecules is a complex and not completely understood topic. Although the primary sequence of different silk fibroins varies meaningfully based on properties and function, natural silk fibroin is an entirely well-defined macromolecule produced virtually without variation from protein to protein. This level of sequence control is currently impossible to achieve at a reasonable cost with synthetic copolymers of even modest molecular weight. Scalability trades off with control in chemical synthesis. For example, solid or liquid phase peptide synthesis can produce uniform peptide segments with excellent sequence control but requires tedious repetitive steps that utilize large quantities of solvent and reagents. In contrast, NCA ROP and chemoenzymatic synthesis are more cost-effective methods of peptide synthesis but do not provide peptides with perfect sequence control. Unfortunately, this problem is not trivial, as studies of silk-mimetic ALA-PEG copolymers have shown that even a difference of two alanine residues in the rigid “crystalline” segment can significantly influence material properties. To simplify the issue, it is likely that uniformity and sequence control are most important for the β-crystalline peptide segment, as dispersity may particularly hinder hydrogen bonding and crystallization. This hypothesis is consistent with general findings on the effect of non-uniformity in synthetic block copolymers [130,131,132] and thermoplastic elastomers [133,134,135]. For example, segmented polyurethanes with uniform rigid blocks have been shown to exhibit increased modulus, tensile strength, and elongation at break [136,137]. Furthermore, dispersity in overall macromolecular size may affect mechanical properties, as short and long chains can have different supramolecular connectivity in an elastomeric network, and thus requires further examination. 

In conclusion, we offer a few conjectures regarding the future of chemical synthesis and application of silk-mimetic materials. To overcome the synthetic challenges described above, including the difficulties in achieving polymers that have high molecular weight, sufficient sequence control, and good solubility for ease of processing, new chemistries and more complex synthesis strategies will be developed. While step-growth polymerization will continue to be utilized, we envision that highly efficient coupling methods, such as “click” chemistry and microwave-assisted polymerization, will be explored to maximize polymer molecular weight. Additionally, incorporating functional groups that protect the polymer from aggregation until removed by outside stimuli, thereby triggering polymer self-assembly, may be implemented as solutions to problems caused by uncontrolled aggregation. Leveraging modern synthesis equipment, which can provide automated and parallel capabilities, libraries of silk-mimetic polymers with systematic variations in dispersity, sequence, and chemical structure can be produced at small scales. Methods developed in recent decades for micro and nano-mechanical testing of materials properties will then enable a more thorough understanding of structure–property relationships in silk-mimetic materials and allow targeted design of such materials for specific applications. These efforts towards targeted synthesis will likely be supported by multi-scale computational simulations that further help predict material properties. Lastly, the potential ability to incorporate non-natural components by chemical synthesis, such as electrically conductive polymer backbones, specific adhesive or recognition motifs, and motifs that respond to light or chemical analytes, can expand the already broad applications of silk-mimetic polymers to electronic sensors and devices, membranes for separation and capture, dynamic actuating materials, and other materials with unique properties. 

## Figures and Tables

**Table 1:** Mechanical property comparison of silk, natural, and synthetic fibers. Data obtained from ref. [3].

Material	Density (g/cm^3^)	Elongation at Break (%)	Strength (GPa)	Young’s Modulus (GPa)	Toughness (MJ/m^3^)
Dragline Silk, *A. diadematus*	1.3	27	1.1	10	180
Flag Silk, *A. diadematus*	1.3	270	0.5	0.003	150
Cocoon Silk, *B. mori*	1.3	18	0.6	7	70
Steel	7.8	0.8	1.5	200	6
Elastin	1.3	15	0.002	0.001	2
Carbon Fiber	1.8	1.3	4	300	25
Kevlar 49	1.4	2.7	3.6	130	50
Nylon 6,6	1.1	18	0.95	5	80
Wool (at 100% relative humidity)	1.3	50	0.2	0.5	60

**Table 2:** Peptide motifs sequences commonly found in various silk fibroins.

Silk Fibroin 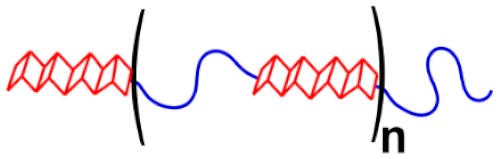	Rigid Segment Motifs (β-Sheet Forming) 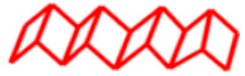	Flexible Segment Motifs 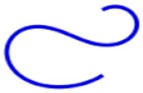
*A. diadematus,* dragline MaSp1 [54,55,56]	(A)_n_	GGX
*A. diadematus,* dragline MaSp2 [54,55,56]	(A)_n_	GPGXX, GGX
*B. mori,* cocoon [45]	(GA)_n_	GY, GV
*A. diadematus,* flagelliform [57,58]	n/a *	GPGXX, GGX
*E. variegata,* cocoon [59]	(A)_9_E(A)_12_, (GA)_n_	GGY, GSG

* β-sheet forming motifs are generally absent in flagelliform silk.

**Table 3:** Known solubilities of silk-mimetic polymers discussed in this work in tetrahydrofuran (THF), N-methyl-2-pyrrolidone (NMP), dimethylformamide (DMF), dimethylsulfoxide (DMSO), dimethylacetamide (DMAC), formic acid (FA), chloroform (CHCl_3_), dichloroacetic acid (DCA), trifluoroacetic acid (TFA), hexafluoroisopropanol (HFIP), trifluoroethanol (TFE), and propylene glycol monoether acetate (PGMEA). Only positively confirmed solubilities are shown; blank spaces indicate unknown solubility.

Polymer	THF	NMP	DMF	DMSO	DMAC	CHCl_3_	DCA	TFA	HFIP	TFE	PGMEA
GAGA-PEG [97]		+		+			+			+	
ALA-PEG [96]							+		+		
(A)_5_-PI [101]						+ ^a^					
GAGA-pTHF [107,108]	+		+	+							
PBLG-PTMEG [110]			+						+		
ALA-PCL [112]				+				+			
(A)_x_-(G-*r*-L)_y_ [117]		+			+						
poly(APA-*b*-MA) [92]				+							+
AN-*co*-silk fibroin [128]			+ ^b^								

^a^ (A)_5_-PI is soluble in a mixture of CHCl_3_ with ClCH_2_CH_2_OH; ^b^ AN-*co*-silk fibroin is not soluble in DMF with silk fibroin content of 0.065 or above.
